# Computer Visual Syndrome in Medical Students From a Private University in Paraguay: A Survey Study

**DOI:** 10.3389/fpubh.2022.935405

**Published:** 2022-07-14

**Authors:** Johanna Coronel-Ocampos, Jonathan Gómez, Alexis Gómez, Pedro P. Quiroga-Castañeda, Mario J. Valladares-Garrido

**Affiliations:** ^1^School of Medicine, Universidad del Pacífico, Asunción, Paraguay; ^2^School of Medicine, Universidad de San Martín de Porres, Chiclayo, Peru; ^3^Vice-rectorate for Research, Universidad Norbert Wiener, Lima, Peru; ^4^Office of Epidemiology, Hospital Regional Lambayeque, Chiclayo, Peru; ^5^Directorate of Health Research, Instituto de Evaluación de Tecnologías en Salud e Investigación-IETSI, EsSalud, Lima, Peru

**Keywords:** medical students, visual fatigue, computer visual syndrome, eye disorder, Paraguay

## Abstract

**Purpose:**

To determine the prevalence and factors associated with computer vision syndrome in medical students at a private university in Paraguay.

**Methods:**

A survey study was conducted in 2021 in a sample of 228 medical students from the Universidad del Pacífico, Paraguay. The dependent variable was CVS, measured with the Computer Visual Syndrome Questionnaire (CVS-Q). Its association with covariates (hours of daily use of notebook, smartphone, tablet and PC, taking breaks when using equipment, use of preventive visual measures, use of glasses, etc.) was examined.

**Results:**

The mean age was 22.3 years and 71.5% were women. CVS was present in 82.5% of participants. Higher prevalence of CVS was associated with wearing a framed lens (PR = 1.11, 95% CI: 1.03–1.20). In contrast, taking a break when using electronic equipment at least every 20 min and every 1 h reduced 7% (PR = 0.93, 95% CI: 0.87–0.99) and 6% (PR = 0.94, 95% CI: 0.89–0.99) the prevalence of CVS, respectively.

**Conclusion:**

Eight out of 10 students experienced CVS during the COVID-19 pandemic. The use of framed lenses increased the presence of CVS, while taking breaks when using electronic equipment at least every 20 min and every 1 h reduced CVS.

## Introduction

Computer vision syndrome (CVS) is defined as “a group of visual and ocular problems related to the prolonged use of computers and devices with video terminals” (1). This syndrome is determined by three mechanisms: the extra ocular mechanism, due to poor posture in front of computer devices, causing musculoskeletal symptoms; accommodative mechanism produces blurred vision, diplopia, myopia and delay in the change of focus; and finally, the ocular surface mechanism, which is related to corneal dryness, reduced blink rate, increased corneal exposure caused by horizontal gaze at the screen of computing devices (2–5). Currently it is estimated that there are 60 million people who suffer from CVS (6), this is due to the fact that today the hours spent in front of an electronic screen are more constant, in order to meet the demands of the modern world (7). Studies report that spending more than 3 h a day in front of video terminal devices increases the prevalence of CVS (8, 9).

In China, 50.8% and 74.3% of Chinese and foreign medical students; respectively, presented CVS. In Saudi Arabia, a prevalence of 97.3% was found for CVS; in turn, the most frequent symptoms were headache, sensation of affected vision (myopia or hyperopia), itchy eyes and burning sensation (10). In Jamaica, 40.3% of students used the computer for more than 6 h and the most frequent symptoms associated with CVS were neck pain, eyestrain, shoulder pain and burning eyes (11). Another study concluded that pre-existing eye diseases (myopia) had a significant relationship with computer-related visual symptoms (12). In Peru, 61% of postgraduate students presented CVS, in turn those who suffered from myopia and astigmatism had a prevalence of CVS of 36.1% and 24.6%; respectively (13). In Peruvian medical students, in the context of the COVID-19 pandemic, the prevalence of CVS was 80.6%. Male sex, age between 16–23 years, time spent of more than 6 h a day in front of the computer or more than 5 h in front of the cell phone, the use of preventive visual measures and not having eye diseases was associated with CVS (14).

The CVS represents a public health problem (15), mainly in medical students, due to their high frequency of use of computer devices (16–19), even more so in the current context of the COVID-19 pandemic, where they have increased their use for virtual education (14, 20–23). However, few studies have evaluated the impact of virtual education on the development of CVS during the COVID-19 pandemic (20, 21), much less in Latin-America (14). Additionally, the influence of hour of device use per day (notebook, smartphone, PC, tablet) on the presence of CVS has not been explored, which is addressed in this research.

Therefore, the objective of this study is to determine the prevalence and factors associated with CVS in medical students at a university in Paraguay.

## Materials and Methods

### Study Design, Population and Sample

A cross-sectional survey study was conducted in medical students from the Universidad del Pacífico, Paraguay during the period from July to September 2021. For the calculation of the sample size, an expected frequency of 80%, a confidence interval of 95% and a precision of 5% was estimated for a population size of 584 students. A sample size of 174 was calculated, of whom 254 were invited to the study and only 228 accepted, representing 39% of the total. Non-probabilistic convenience sampling was used. Students who completed the variables of interest in the study were included. Students who did not wish to participate in the survey were excluded.

### Variables

The dependent variable was CVS, operationally defined as the presence of at least one symptom such as eye fatigue, blurred vision, headache, dry eyes, excessive tearing, blurred vision, double vision, eye irritation, and changes in color vision; that occur due to the use of a computer or other electronic device, either regular or frequent, in at least 1 week of the last 12 months (24).

The independent variables were age, gender, academic block, glasses, hours spent on the notebook, hours spent using the smartphone, use of visual preventive measures, taking breaks when using computers, and previous eye diseases.

### Measures

A questionnaire consisting of 2 sections was designed (see Supplementary File 1).

Socio-educational data: a questionnaire was constructed based on 9 closed questions, in order to obtain information in a general way from each student surveyed. This questionnaire included: age (expressed in years), sex (male or female), academic cycle (clinical or preclinical), use of glasses (not wearing, glasses with frames or contact lenses), hours of use per day of notebook, Smartphone, PC and tablet (<2 h, 2–4 h, 4–6 h, more than 6 h), use of visual preventive measures (Yes or No), taking a break after using equipment (at least every 20 min, every 1 h, every 2 h, after more than 2 h, no rest), eye diseases (Yes or No).

CVS Questionnaire (CVS-Q): Designed to measure the CVS, it is composed of 16 items, which are the symptoms perceived by the student, in order to evaluate the frequency with its categories of: never (none occasion), occasionally (eventually or once a week), often or always (2 or 3 times a week or almost every day); and intensity with its categories: Moderate or Intense. The values for the categories are as follows: Never = 0 points, Occasionally = 1 point, Often or always = 2 points, Moderate = 1 point, and Severe = 2 points. The result of the multiplication of frequency and intensity will give us the severity that must be recoded with the following values: 0 = 0 points, 1 or 2 = 1 point, 4 = 2 points; and then the severity scores are added, and if they yield a score ≧ 6 points, the student will be considered to have a computer vision syndrome. It should also be noted that if the student never marked the frequency, nothing would be marked in the intensity box. This questionnaire presents the following psychometric properties: Cronbach's alpha of 0.87 and a sensitivity and specificity > 70%. With which it becomes an acceptable questionnaire (25).

### Procedures

A virtual questionnaire was created using the Google Forms tool to collect the data. The virtual link of the survey was socialized through social networks (email, WhatsApp groups) of each academic year. After collecting the data, they were processed using the Microsoft Excel program.

### Data Analysis

A univariate analysis was performed, which consisted of frequencies (relative and absolute), measures of central tendency and dispersion (mean and standard deviation, median and interquartile range). The factors associated with CVS were evaluated using the chi-square test, after evaluating the assumption of expected frequencies. In the case of the numerical variable (Age), the student's *t*-test was used, after evaluating the assumption of distribution in both comparison groups. *p* < 0.05 was considered as statistically significant.

Simple and multiple regression models were built, and prevalence ratios (PR) and 95% confidence intervals (95% CI) were estimated to evaluate the potential factors associated with CVS. Poisson distribution family, log link function and robust variance were used.

Statistical analysis was performed in the Stata v.17.0 program.

### Ethical Considerations

The research was reviewed and approved by the Ethics Committee of the Universidad del Pacífico, Paraguay. The confidentiality of the participants was preserved at all times, since the questionnaire was anonymous. Informed consent was requested prior to study participation. The principles of the Declaration of Helsinki (beneficence, non-maleficence, autonomy, justice and confidentiality) were considered.

## Results

Of 228 medical students surveyed, the mean age was 22.3 years, 71.5% were women, 52.2% were in preclinical academic cycles. Most of the participants (61.8%) reported having a previous ocular disease and 59.2% reported wearing framed lenses. 12.3% of students reported using the computer/laptop for more than 6 h. 70.2% of students reported using the smartphone for more than 6 h. 25.4% reported taking breaks after more than 2 h of technology use. 82.5% presented SVI. Table 1

**Table 1:** Characteristics of participants and use of technologies (*n* = 228).

**Characteristics**	***N*** **(%)**
Age (years)*	**22.3** **±2.6**
Sex	
Female	163 (71.5)
Male	65 (28.5)
Cycle	
Preclinical	119 (52.2)
Clinical	109 (47.8)
Previous ocular disease	
No	87 (38.2)
Yes	141 (61.8)
Use of lenses	
No	86 (37.7)
With frame	135 (59.2)
Contact lenses	7 (3.1)
Use of preventive visual measures	
No	42 (18.4)
Yes	186 (81.6)
Hours in the day spent using a notebook	
<2 h	28 (12.3)
Between 2 to 4 h	38 (16.7)
Between 4 to 6 h	50 (21.9)
More than 6 h	112 (49.1)
Hours in the day spent using a smartphone	
<2 h	10 (4.4)
Between 2 to 4 h	15 (6.6)
Between 4 to 6 h	43 (18.9)
More than 6 h	160 (70.2)
Hours in the day spent using a tablet	
<2 h	157 (68.9)
Between 2 to 4 h	28 (12.3)
Between 4 to 6 h	19 (8.3)
More than 6 h	24 (10.5)
Hours in the day spent using a PC/Laptop	
<2 h	171 (75.0)
Between 2 to 4 h	14 (6.1)
Between 4 to 6 h	15 (6.6)
More than 6 h	28 (12.3)
Breaks taken of technology use	
Don‘t take breaks	36 (15.8)
At least every 20 min	57 (25.0)
Every 1 h	49 (21.5)
Every 2 h	28 (12.3)
After more than 2 h	58 (25.4)
CVS**	
Absent	40 (17.5)
Present	188 (82.5)

*Mean ± standard deviation **CVS, Computer visual syndrome.

Students who had previous ocular disease had an 18.1% higher prevalence of CVS, compared to those without disease (89.4% vs. 71.3%; *p* <0.001). Students who took preventive visual measures had a 16.4% higher prevalence of CVS, compared to those who did not take any measures (85.5% vs. 69.1%; *p* = 0.011). Additionally, the use of glasses (*p* < 0.001), hours of daily tablet use (*p* = 0.015) and taking breaks when using electronic equipment (*p* = 0.002) were associated with CVS Table 2.

**Table 2:** Factors associated with computer visual syndrome.

**Variables**		***CVS***		p*
		**No (*n* = 40)**	**Yes (*n* = 188)**	
		***n*** **(%)**	***n*** **(%)**	
Age (years)***		22.5 ± 2.1	22.3 ± 2.8	0.635**
Sex				0.317
	Female	26 (16.0)	137 (84.1)	
	Male	14 (21.5)	51 (78.5)	
Previous ocular disease				**<0.001**
	No	25 (28.7)	62 (71.3)	
	Yes	15 (10.6)	126 (89.4)	
Use of lenses				**<0.001**
	No	26 (30.2)	60 (69.8)	
	With frame	12 (8.9)	123 (91.1)	
	Contact lenses	2 (28.6)	5 (71.4)	
Use of preventive visual measures				**0.011**
	No	13 (31.0)	29 (69.1)	
	Yes	27 (14.5)	159 (85.5)	
Hour in the day spent using a notebook				0.090
	≤ 4 h	16 (24.2)	50 (75.8)	
	≥ 4 h	24 (14.8)	138 (85.2)	
Hour in the day spent using a smartphone				0.830
	≤ 4 h	4 (16.0)	21 (84.0)	
	≥ 4 h	36 (17.7)	167 (82.3)	
Hour in the day spent using a tablet				**0.015**
	≤ 4 h	27 (14.6)	158 (85.4)	
	≥ 4 h	13 (30.2)	30 (69.8)	
Hour in the day spent using a PC/Laptop				0.517
	≤ 4 h	31 (16.8)	154 (83.2)	
	≥ 4 h	9 (20.9)	34 (79.1)	
Breaks taken of technology use				**0.002**
	Don‘t take breaks	4 (11.1)	32 (88.9)	
	At least every 20 min	15 (26.3)	42 (73.7)	
	Every 1 h	10 (20.4)	39 (79.6)	
	Every 2 h	9 (32.1)	19 (67.9)	
	After more than 2 h	2 (3.5)	56 (96.6)	

*p-value of categorical variables calculated with the Chi Square test.

**p-value of categorical–numerical variables calculated with the Student's t-test.

***Mean ± standard deviation.

*Significant p-values (p < 0.05) are highlighted in bold*.

**Figure 1:**
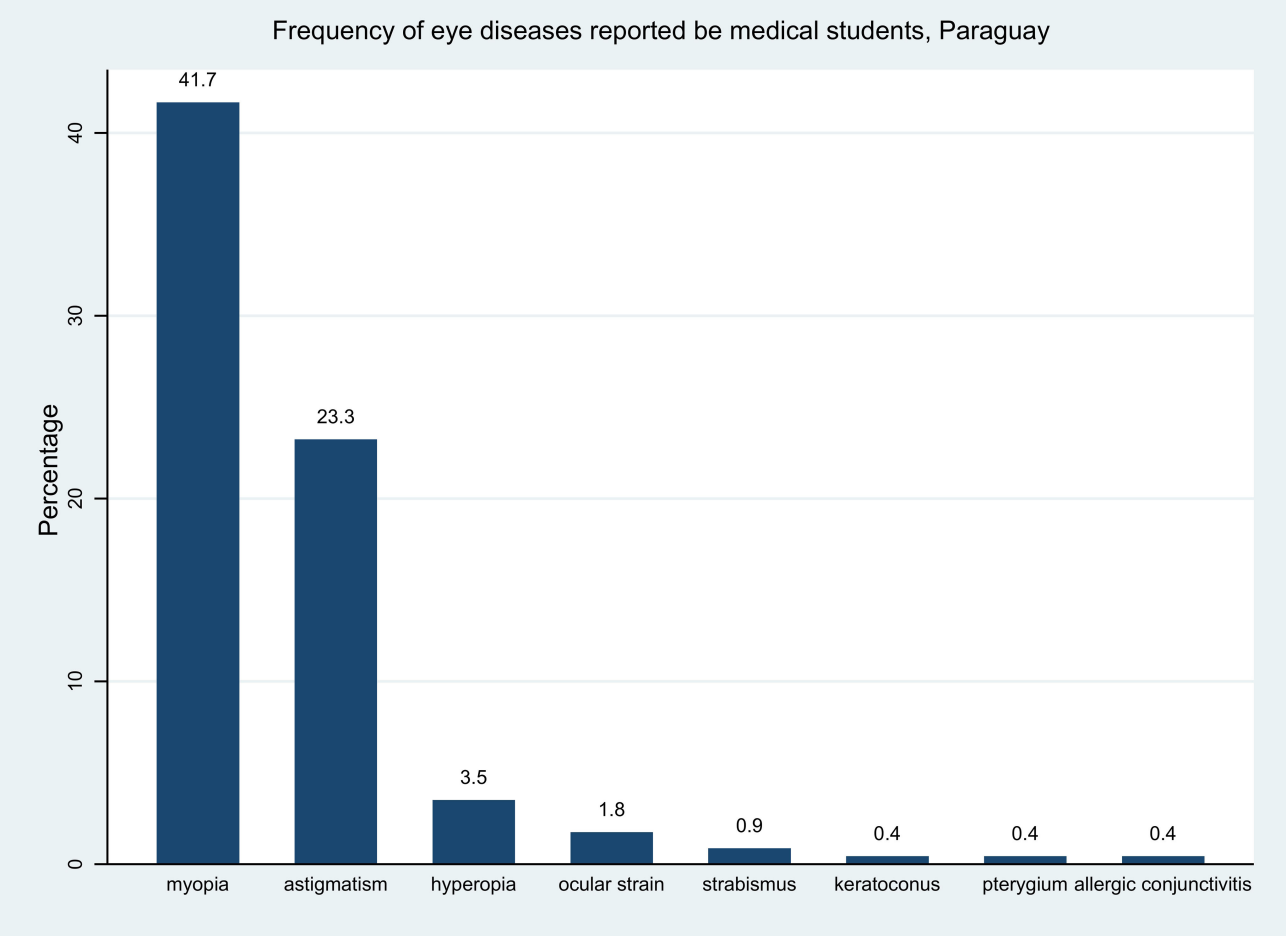
Frequency of self-reported eye diseases.

The eye diseases that the students reported most frequently were myopia (41.7%) and astigmatism (23.3%) Figure 1.

In the simple model, we found that the factors associated with a higher prevalence of CVS were, having previous ocular disease (PR = 1.11; 95% CI: 1.02–1.20), wearing framed lenses (PR = 1.13; 95% CI: 1.04–1.22), use of a notebook more than 4 h a day (PR = 1.05; CI95%: 1.01–1.11). On the contrary, the factors associated with a lower prevalence of CVS were taking a break at least every 20 min (PR=0.92; CI95%:0.86–0.98) and every 2 h (PR = 0.89; CI95%:0.80–0.99) when electronic equipment is used. In the multiple models, we found that students who wear framed lenses had an 11% higher prevalence of CVS (PR = 1.11; 95% CI: 1.03–1.20). Taking breaks when using electronic equipment at least every 20 min and every 1 h reduces the prevalence of CVS by 7% (PR = 0.93; 95% CI:0.87–0.99) and 6% (PR = 0.94; 95% CI:0.89–0.99); respectively Table 3.

**Table 3:** Factors independently associated with computer visual syndrome, simple and multiple regression analysis.

**Characteristics**		***CVS***					
		**Simple regression**			**Multiple regression**		
		**PR**	**95% CI**	p*	**PR**	**95% CI**	p*
Age (years)			1.00	0.99–1.01	0.694		
Sex							
Female		Ref.					
Male		0.97	0.89–1.05	0.473			
Previous ocular disease**							
No		Ref.					
Yes		1.11	1.02–1.20	**0.015**			
Use of lenses							
No		Ref.			Ref.		
With frames		1.13	1.04–1.22	**0.006**	1.11	1.03–1.20	**0.007**
Contact lenses		1.01	0.84–1.22	0.918	0.98	0.86–1.11	0.706
Use of preventive visual measures							
No		Ref.					
Yes		1.10	0.96–1.25	0.173			
Hour in the day spent using a notebook							
≤ 4 h		Ref.			Ref.		
≥ 4 h		1.05	1.01–1.11	**0.049**	1.02	0.99–1.06	0.235
Hour in the day spent using a smartphone							
≤ 4 h		Ref.					
≥ 4 h		0.99	0.93–1.05	0.767			
Hour in the day spent using a tablet							
≤ 4 h		Ref.					
≥ 4 h		0.92	0.82–1.02	0.117			
Hour in the day spent using a PC/Laptop							
≤ 4 h		Ref.					
≥ 4 h		0.98	0.92–1.04	0.472			
Breaks taken of technology use							
Don‘t take breaks		Ref.			Ref.		
At least every 20 min		0.92	0.86–0.98	**0.014**	0.93	0.87–0.99	**0.019**
Every 1 h	0.95		0.90–1.00	0.073	0.94	0.89–0.99	**0.019**
Every 2 h	0.89		0.80–0.99	**0.032**	0.91	0.81–1.02	0.100
After more than 2 h		1.04	0.96–1.13	0.327	1.04	0.97–1.11	0.313

*p–values obtained with Generalized Linear Models, Poisson family, log link function, robust variance.

**Does not enter multiple model due to collinearity.

*PR, prevalence ratio; 95% CI, 95% confidence interval*.

*Significant p-values (p < 0.05) are highlighted in bold*.

## Discussion

### Prevalence of Computer Visual Syndrome

CVS represents a medical condition which risk has increased exponentially after the implementation of the new public health measures to prevent the transmission of SARS-CoV-2, by suspending face-to-face university activities, exposure to devices has increased. Computer systems, which inevitably represents negative effects at the ocular/visual level (14). In the present study, it was obtained the participation of 228 Medicine students who took hybrid classes, meaning, face-to-face and virtual classes, at a private university in Paraguay.

We found that almost 8 out of 10 students presented CVS (82.5%). This is higher than what was found in Chinese medical students during the SARS-CoV-2 outbreak period, where the prevalence of CVS was 50.8% and 74.3% in Chinese and foreign students; respectively (21). In a study in which the CVS-Q was used in medical university students in Spain, the prevalence was 76.6%, somewhat lower compared to our study (82.5%) (26). A study conducted during virtual education due to the COVID-19 pandemic in Lima, Peru in medical students applying the CVS-Q, estimated a slightly lower prevalence compared to our study (80.6%) (14). However, the prevalence from this study was similar with pre-pandemic estimates. A study in Italian engineering students found that 90% of them presented visual fatigue during long sessions of computer-aided design (27). Also, a study in Malaysian university students showed that 89.9% of them presented CVS (28). Conversely, in office workers, a lower prevalence (approximately 50 to 70%) was reported (4, 5). These differences may be due in part to the different use demands of computers or electronic devices in certain careers, which makes CVS an important feature in students. Also, in the case of work offices, preventive measures may have been timely implemented, such as intermittent breaks during work hours. This contrasts with the medical students' context, in which they are mostly exposed to long and continuous hours of study and therefore potential greater exposure to electronic devices (29). The prevalence reported here allows us to highlight the problem that the pandemic have caused in ocular health of medical students, and how it would shape up in the long term. This inadvertent exposure in medical students would bring health sequelae that we intend to alert the authorities of local and regional medical schools.

Additionally, we found that almost half of the students reported having myopia. This is similar to what was reported in Peru, where almost half (36.1%) of postgraduate students presented myopia (30). However, it differs from what was reported in Arabia, where myopia was one of the most significant findings related to CVS, being found in more than half of the students (77.8%) (12). However, it differs from what was reported in Chinese schoolchildren during the COVID-19 pandemic, where almost half (48%) presented eye dryness and itching (20).

### Factors Associated With Computer Visual Syndrome

According to the multiple regression analysis, students who took breaks at least every 20 min and every 1 h reduced the prevalence of having CVS. This is similar to multiple studies conducted in Arabia and Peru reported that taking rest is considered a protective factor for CVS (14, 31). In 2,363 students from China, failure to take a 20-sec break every 20 min of work was found to increase the likelihood of having CVS during the COVID-19 pandemic (20). This association could be explained because it has been seen that taking breaks, whether short or long, contributes to the relaxation of the eye muscles, thus preventing eye fatigue or tiredness (6).

In our study, multivariate analysis showed that those who wear framed lenses have a higher prevalence of suffering from CVS. This contrasts with another study carried out in Peru, in which no significant association was found. On the contrary, other works, such as a study in India, coincide with our reports, where it was found that the use of framed lenses and having blurred vision increase the frequency of CVS. The discrepancy between the studies mentioned could be due to the fact that the use or not of certain protective filters in said lenses was not included in the instrument, generating information bias in the investigations carried out (10, 14, 32).

A noteworthy result from the simple regression analysis showed that students who reported having a history of some ocular pathology had an 11% higher prevalence of CVS, which coincides with other studies reported in Peru and Saudi Arabia (12, 14). This is consistent with what was described by a similar study, where it was found that medical students who had previous ocular pathologies such as myopia had a significant relationship with presenting CVS (12). In China, research conducted in schoolchildren during virtual learning in the COVID-19 pandemic found that those with other ocular diseases were more likely to have CV (20). However, it contrasts with another study carried out in Saudi Arabia, where it was found that refractive errors such as myopia and hyperopia did not have a significant association with CVS (26). The possible explanation for this association is that due to the long hours that the student spends in front of a computer, a greater sustained effort is needed for visual accommodation and this leads to the generation of ocular discomfort such as a sensation of tearing, blurred vision and other symptoms that make up CVS (16).

In this study, we have found that students used mostly smartphones compared with other electronic devices. This higher use was not supported by the bivariate and multivariate analysis to demonstrate that this behavior increased the prevalence of perceived CVS. This could be explained by the low sample size used to compare the prevalence estimates of CVS according to the time spent on smartphones. However, we did not collect additional information on which device was mainly used for classes or study. This limits our strength to assume that higher smartphone use increased the prevalence of CVS. Another possible reason for this absent difference is that students may have used electronic devices with time breaks for exposure reduction. This particular use may be related to the awareness they had with ocular health. In addition, a cutoff of 4 h to compare the use patterns of electronic devices might not be precise enough to find differences on the prevalence of CVS (33–35).

### Limitations and Strengths

This research has some limitations. First, potential selection bias, since they only involve findings from one university site, therefore, the results cannot be inferred to the entire study population. Second, due to the cross-sectional design of the study, it is not possible to attribute causality between the variables that were associated with CVS. Third, it was not possible to measure other confounding variables for CVS such as stress, hours of sleep, presence of dry eye, which could lead to measurement bias. Despite the aforementioned limitations, an instrument with optimal psychometric properties has been used to measure CVS and represents a solid approach on the factors influencing CVS in physicians in training in Latin America during the COVID-19 pandemic.

### Public Health Implications of Findings

The findings of this study provide us with preliminary scientific evidence that CVS is very prevalent in human medicine students. Therefore, it provides a consistent analysis to be able to use corrective measures and thus be able to avoid progression to CVS; in turn, avoid generating higher expenses in treatments caused by this syndrome and optimize the student's academic performance. Also encourage other studies to be carried out with greater rigor (in which a follow-up is carried out throughout the different academic cycles, incorporating more university populations from different academic faculties, incorporating other variables that could be associated with CVS such as dry eye, stress, hours of sleep, among others), it would also be extremely important that the research contains at least one group of specialists in the field, and thus reach conclusions that can contribute satisfactorily in the study of this pathology.

### Conclusions

This study determined the prevalence of CVS and its associated factors in medical students from a Paraguayan university. We found that 8 out of 10 students had CVS during the COVID-19 pandemic. Taking breaks when using electronic equipment at least every 20 min and every 1 h reduces the prevalence of CVS. Conversely, the use of framed lenses increases this condition. Our findings highlight the importance of preventing CVS in medical students, encouraging the use of technology in an early and timely manner. Special attention should be paid to those presenting the risk factors described here, to reduce complication related to CVS.

## Data Availability Statement

The raw data supporting the conclusions of this article will be made available by the authors, without undue reservation.

## Ethics Statement

The studies involving human participants were reviewed and approved by Ethics Committee of the Universidad del Pacífico, Paraguay. The patients/participants provided their written informed consent to participate in this study.

## Author Contributions

JC-O was involved with conceptualization. JG and PQ-C conducted literature searches. JC-O, JG, and AG wrote original draft of the manuscript and were involved with formal analysis. MV-G gave input on specification of models and methodology. JC-O and MV-G verified the underlying data and curated data. All authors contributed to conceptualization, data interpretation, and manuscript review and editing. All authors contributed to the article and approved the submitted version.

## Conflict of Interest

The authors declare that the research was conducted in the absence of any commercial or financial relationships that could be construed as a potential conflict of interest.

## Publisher's Note

All claims expressed in this article are solely those of the authors and do not necessarily represent those of their affiliated organizations, or those of the publisher, the editors and the reviewers. Any product that may be evaluated in this article, or claim that may be made by its manufacturer, is not guaranteed or endorsed by the publisher.
