# Supplementary material for: Computer Visual Syndrome in Medical Students From a Private University in Paraguay: A Survey Study
Source: Front Public Health. 2022 Jul 14;10:935405. doi: 10.3389/fpubh.2022.935405 (PMC9330381; doi:10.3389/fpubh.2022.935405)
Supplement: Supplementary file 1 [file Table_1.DOC]

Síndrome visual informático

El síndrome visual informático (SVI) o Computer vision syndrome (SCV) es definido por la Asociación Americana de Optometría como “un grupo de problemas visuales y oculares relacionados al uso prolongado de computadoras y dispositivos con video terminales.

Los datos recabados en la presente encuesta serán utilizados en la redacción de un artículo científico, para el cuál no se recolectarán datos personales de los participantes; es decir, la encuesta es completamente anónima.

*Obligatorio


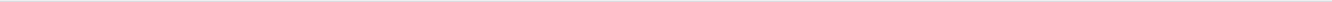


1. Acepta ud. participar de la encuesta?

*

*Marca solo un óvalo.*


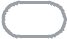
 Sí *Ir a la pregunta 2*


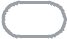
 No

1. Sexo

*

*Marca solo un óvalo.*


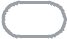
 Masculino


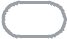
 Femenino

1. Edad (en números)

*


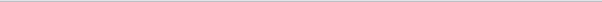


1. Año de carrera

*

*Marca solo un óvalo.*


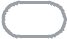
 1°


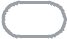
 2°


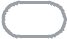
 3°


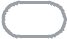
 4°


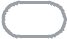
 5°

6°

1. Con qué frecuencia utiliza los siguientes aparatos informáticos ?

*

*Selecciona todas las opciones que correspondan.*

| Menos de 2 | Entre 2 y 4 | Entre 4 y 6 | Más de | 6 |
| --- | --- | --- | --- | --- |
| horas | horas | horas | horas |  |
|  |  |  |  |  |

**Tab**l**et**

**
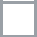

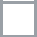

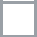

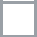

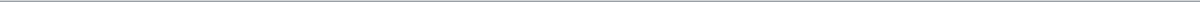
**

**Notebook**

**
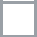

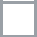

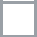

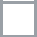

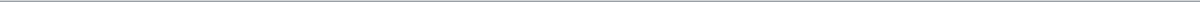
**

**Smartphone**

**
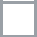

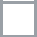

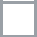

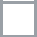

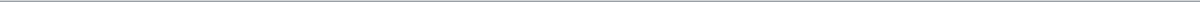
**

**Computador de**

**
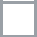

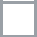

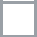

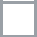
**

**mesa**

**
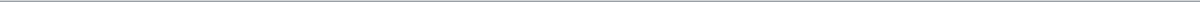
**

1. Toma descansos durante el uso de aparatos informáticos?

*

*Marca solo un óvalo.*


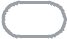
 Almenos cada 20 minutos


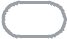
 Cada 1 hora


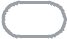
 Cada 2 horas


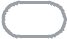
 Después de más de 2 horas


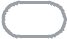
 No toma descansos

1. Uso de medidas visuales preventivas?

*

*Selecciona todas las opciones que correspondan.*

*
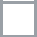
*

No tomo ninguna medida preventiva


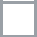


Uso de lágrimas artificiales


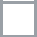


Fijar la mirada a sitios lejanos


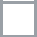


Mantener los ojos cerrados por un tiempo


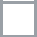


Uso de lentes


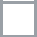


Regular brillo de pantalla

1. Marque la opción e intensidad que considere correcta según sus síntomas oculares

*

*Selecciona todas las opciones que correspondan.*

a

menudo Intensidad Intensidad

nunca ocasionalmente

o moderada alta

siempre


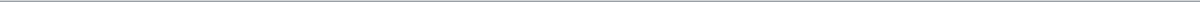


**Ardor ocu**l**ar**

**
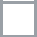

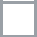

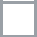

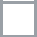

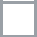

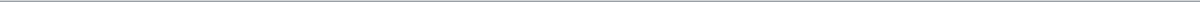
**

**p**i**cor ocu**l**ar**

**
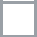

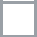

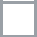

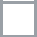

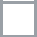

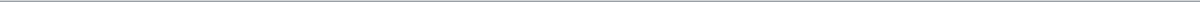
**

**Sensac**i**ón de cuerpo**

**
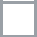

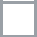

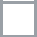

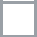

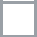
**

**extraño**

**
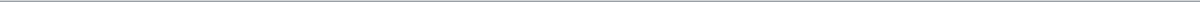
**

**Parpadeo exces**i**vo**

**
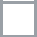

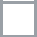

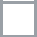

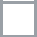

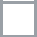

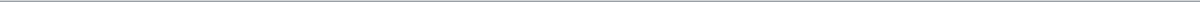
**

l**agr**i**meo**

**
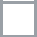

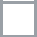

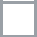

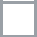

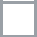

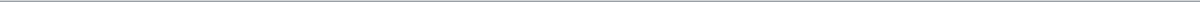
**

**Do**l**or ocu**l**ar**

**
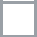

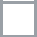

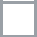

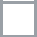

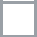

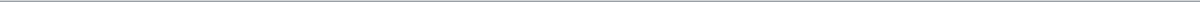
**

**Sequedad**

**
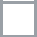

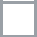

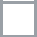

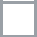

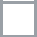

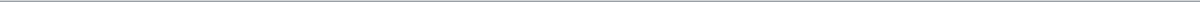
**

**V**i**s**i**ón borrosa**

**
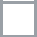

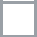

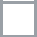

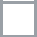

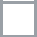

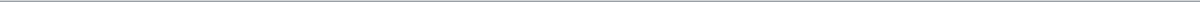
**

**V**i**s**i**ón dob**l**e**

**
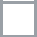

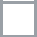

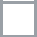

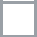

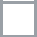

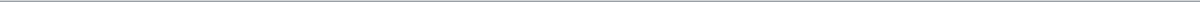
**

**D**i**f**i**cu**l**tad a**l **enfocar**

**
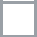

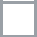

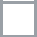
**

**en v**i**s**i**ón de cerca**

**Aumento de**

**sens**i**b**ili**dad a** l**a** l**uz**

**Ha**l**os de co**l**ores**

**a**l**rededor de** l**os**

**ob**j**etos**

**Sensac**i**ón de ver peor**

**Do**l**or de cabeza**

**Do**l**or Cerv**i**ca**l

1. Utiliza lentes de graduación?

*

*Marca solo un óvalo.*

No

Con marco

De contacto

1. Presenta alguna enfermedad ocular diagnosticada previamente?

*

*Marca solo un óvalo.*

Sí

No

1. De haber contestado positivamente la pregunta anterior, marque la enfermedad ocular que posee

*Selecciona todas las opciones que correspondan.*

Miopía

Astigmatismo

Presbicia

Hipermetropía

Otros:

Google no creó ni aprobó este contenido.

[Formularios](https://www.google.com/forms/about/?utm_source=product&utm_medium=forms_logo&utm_campaign=forms)
